# Professional Etiquette

**Published:** 1853-10

**Authors:** 


### ARTICLE X.

### *Professional Etiquette.*

My Dear Doctor,—May "a friend in difficulties" take the liberty of applying to you in a dilemma, to her most embarrassing and unaccountable; and can you spare a moment of your valuable time to give the desired information? Your well known devotion to the gentler sex, assures me that I will not be overlooked merely because I am a woman, but I know you have so many other more important matters to attend to, that I shall esteem it a great favor, if, in the press of business, I am not altogether overlooked. Trusting, therefore, that some day my feminine appeal may be remembered, I will proceed at once to state the difficulty I have encountered, well convinced that if any one can explain the matter, it must be yourself.

Fortunately for me, dear doctor, I have so far altogether escaped the delightful sensation of taking my first seat in a dentist's chair; nature, in a liberal mood, having provided me with so perfect a set of masticators, that I have been altogether independent of your skill, and able to look on forceps, pullikins, lancets, and all the keen paraphernalia of your private inquisition, with a coolness and composure quite aggravating to those of less happy experience. No little blue bright blade, with its jeweled handle, and other insolent mockery of ornaments, made me shudder, as it awakened recollections of the past days and hours of torture.

This state of things, however, was too good to be permanent, and a short time since, just on the eve of what may be considered the most important event of my life, I discovered, to my dismay, symptoms of rapid decay in my central incisors. Here was a pretty condition of matters. Those teeth were of incalculable value to me. To say nothing of their usefulness, they had always taken a prominent place in any little compliment addressed to my personal appearance, and indeed, in seasons of low spirits, I was sometimes haunted with the idea, that there might be more truth than flattery in the remark of some ill-natured people, that they were "my only beauty." Certain it was, I could not afford to lose them, and without daring to look that possibility in the face (only think, doctor, what it was—the loss of lover, husband, the wreck of the heart's best affections, then "cold inhumanity, burning insanity," &c., &c., all hanging on two teeth.) I hastened to your city, determined to endure any thing, every thing, rather than lose them.

There, on inquiring for the most eminent dentist, I was, of course, directed to you, and with a firm resolution to "suffer and be strong," I rung your bell. Alack! you had left town on business, and would not be back for "ever so long," so, with "a silent sorrow in my breast," I sought the residence of another practitioner, and he being at home, submitted to a lengthy inspection of my case, and tremblingly awaited his decision. The prognosis was rather favorable, and he named an early hour the next day for the operation. "But," he added, "it is fortunate you came to *me*—not another dentist in the city could save those teeth. No other understands the business. You see I have my own peculiar method, and though these quacks that you may meet with every where, hanging out their impertinent signs, may have temerity enough to attempt any thing, believe me, there is not one amongst them that knows any thing about the teeth." I must confess I was rather startled at this gratuitous piece of information, but left, inwardly congratulating myself on having so fortunately stumbled on the right man.

That evening, however, in a little social circle, I happened to meet another member of your profession, and innocently detailed to him my adventures of the morning, and mentioned my engagement for the next day with Dr. No. 2, as I will call him.

"What!" exclaimed my new acquaintance, perfectly horror-stricken, "you are not going to let that incorrigible ignoramus operate on your teeth? Why he don't understand the first rudiments of his business."

"Oh!" I answered, "he has his own peculiar method," &c.

"His own peculiar *humbug*, Miss; the unconscionable donkey. A pretty sight you will be after you leave his hands;" and

> "There stood the rogue and roared,
> Unasked, and unencored,"

whilst I was ready to cry with vexation.

Of course I determined to leave Dr. No. 2 and take No. 3, since he declared "that it was universally acknowledged by every person capable of forming an opinion on the matter, that *he* alone understood dentistry in all its branches." My troubles, however, were not at an end. The next day, previous to the hour of my appointment with Dr. No. 3, I met a friend in the street, who, as it happened, was on the way to her dentist, and at her earnest solicitation I accompanied her thither, without, however, having any intention of breaking my engagement with Dr. No. 3. But my friend, as soon as we were ushered into the presence of No. 4, thought proper to mention it to him, and I was really startled by the deep resounding groan that burst apparently from the bottom of his heart, when he heard of my rashness and danger."

"Poor child," he ejaculated, shaking his head dismally, "poor child! poor human nature! how constantly it is imposed upon. Oh, it is sad to think how utterly depraved that heart must be, that can deliberately plan and execute an irreparable injury upon a fellow creature. for the sake of a little filthy lucre, the root of all evil. Why, my dear child, that fellow will ruin your teeth to a certainty. What does he know of dentistry? He has not the mind to comprehend it; and if he were to study from now until the age of Methuselah it would make no difference in him. Plugging teeth is an art and a *mystery*, and it takes nothing less than an inborn natural genius to comprehend it. Now believe me, I have no ill-feeling towards Dr. No. 3—no wish to injure him, but as a christian man, I cannot bear to see my fellow creatures imposed upon, and I happen to possess a little specimen of his work which I will show, and you may form your own opinion;" and with this Dr. No. 4 produced from his case, a full set of porcelain teeth, which he declared was an "unusually good specimen of the work of Dr. No. 3," and laid them before us for examination.

Now, doctor, I am, by no means, a judge of these things; but, if those teeth were not the identical set which belonged to poor Dr. Parkman, rescued from the fiery furnace after they had been roasting there for a week, (was it?) they certainly were a fac-simile of the same, and unmistakably, had gone through a similar process. Still, our christian friend, assured us, that he had taken them as they were, from the mouth of one of Dr. No. 3's "victims," and benevolently enlightened us as to any defects that might have escaped our observation. How the "victim" ever got them into his mouth *as they then were*, or being there ever got them out again, must ever remain a mystery to me, and, indeed, if Dr. No. 3, ever did accomplish this, he might turn a pretty penny, by going around the country, and making it a public exhibition for the curious.

Of course, my faith was now shaken in Dr. No. 3, but still, it was by no means established in No. 4. He had overacted his part, and I determined to try again. Here, dear doctor, was but the commencement of my difficulties. Every where I went, and every where I heard the same complaints, the same mutual accusations, the same charges of ignorance, selfishness and cupidity, until I returned home perfectly amazed and disgusted, not knowing what to do, or whom to trust.

Now, doctor, the question is, why is this? What has originated this state of ill-feeling amongst the members of your profession? Other men engaged in the same pursuits, manage to jog along without apparently desiring to disparage all others, or gain a monopoly of custom. Physicians even, do not invariably speak against each other, but often meet in amicable consultation, and suck their canes, and more bedarken a difficult case, in the most good natured obscurity. Even druggists, are not always sworn foes, and can meet and say, "art thou well, my brother?" without a stab under the fifth rib. Then, why is it, that there is so deadly a feud existing between dentists, and why are they so willing to crush one another? I know that many ignorant pretenders have crept into your ranks, men, unwilling to learn, and incapable of understanding their profession, who do incalculable injury in the community, and only deserve contempt and ridicule; but it is not to these I allude. It is to the better classes, and again I ask, why is it?

Yours truly, KATE.

To Dr. C. A. Harris.

*Long Green, September* 15, 1853.

We will reply to our fair friend under the editorial head.

